# The Expression of MTUS1/ATIP and Its Major Isoforms, ATIP1 and ATIP3, in Human Prostate Cancer

**DOI:** 10.3390/cancers3043824

**Published:** 2011-10-11

**Authors:** Simon N.S. Louis, Laurie T.C. Chow, Naghmeh Varghayee, Linda A. Rezmann, Albert G. Frauman, William J. Louis

**Affiliations:** Clinical Pharmacology and Therapeutics Unit, Department of Medicine, University of Melbourne, Austin Health, Heidelberg 3084, Victoria, Australia; E-Mails: ltcchow@gmail.com (L.T.C.C.); naghmehv@hotmail.com (N.V.); l.rezmann@nucleusnetwork.com.au (L.A.R.); albertf@unimelb.edu.au (A.G.F.); slouis944@aol.com (W.J.L.)

**Keywords:** Angiotensin II (Ang II), Ang II type 2 (AT_2_-) receptor, AT_2_-receptor interacting protein (ATIP), prostate cancer, high grade prostatic intraepithelial neoplasia (HGPIN)

## Abstract

Angiotensin II (Ang II), the main effector of the renin angiotensin system, acts upon two distinct transmembrane receptors, the Ang II type 1 and the type 2 (AT_2_-) receptor, to induce promotion and inhibition of ERK2 phosphorylation. The AT_2_-receptor, through an interaction with its putative signaling partner MTUS1/ATIP (AT_2_-receptor interacting protein), inhibits the mitogenic effects of EGF in prostate cancer cell lines representing both early and late stage disease. This is the first report on the expression of ATIP in normal and malignant human prostatic biopsies. The expression of ATIP and its major isoforms, ATIP1 and ATIP3, in normal prostatic cells and three prostate cancer cell lines was examined using QPCR and immunohistochemistry. Human biopsies containing benign prostatic hyperplasia (BPH), high grade prostatic intraepithelial neoplasia (HGPIN) and well, moderately and poorly differentiated prostate cancer were also examined. Overall, ATIP1 and ATIP3 mRNA expression was increased in malignant compared to normal tissues and cell lines. ATIP immunostaining was low or absent in both the basal and columnar epithelial cell layers surrounding BPH acini; however, it was observed in high concentration in neoplastic epithelial cells of HGPIN and was clearly evident in cytoplasms of malignant cells in all prostate cancer grades. ATIP immunostaining was also identified in the cytoplasms of LNCaP and PC3 prostate cancer cells. As the AT_2_-receptor/ATIP inhibitory signaling pathway exists in malignant cells in all grades of prostate cancer, enhancement of this pathway may be a therapeutic target even after the development of androgen-independence.

## 1. Introduction

Recent studies suggest that angiotensin II (Ang II) has a role in the development of prostate cancer and drugs that inhibit Ang II type I (AT_1_-) or stimulate Ang II type 2 (AT_2_-) receptors may modulate the mitogenic effects of Ang II and EGF in prostate cancer [1-4]. ATIP, a putative AT_2_-receptor signaling transducer interacts with the C-terminal tail of the AT_2_-receptor [5-8]. Also known as MTUS1 [9], five different ATIP isoforms have been identified, which are numbered 1, 2, 3A, 3B and 4, and all share a common AT_2_-receptor interacting domain suggesting that all five ATIP isoforms may interact with the AT_2_-receptor [7].

Previously, mRNA for ATIP1, ATIP3a and ATIP3b has been identified in normal human prostate tissue [7]. More recently, we identified ATIP expression in androgen-dependent (LNCaP) and androgen-independent (PC3) prostate cancer cells where we found ATIP1 to be a negative regulator of cell growth acting in concert with the AT_2_-receptor. ATIP1 over-expression reduced basal and EGF mediated ERK phosphorylation in PC3 cells whereas ATIP silencing increased the basal rate of DNA synthesis in PC3 cells and the sensitivity of LNCaP cells to the mitogenic effects of EGF [10].

As ATIP appears to play an important role in human prostate cancer cell growth, we have extended our recent work [10] to determine the expression and localization of ATIP using immunohistochemical techniques in fixed prostate cancer cells and paraffin-embedded human prostatic biopsy materials containing benign prostatic hyperplasia (BPH), high grade prostatic intraepithelial neoplasia (HGPIN), a premalignant state, and a range of prostate cancer grades. In addition, we extended our studies of the expression of ATIP1 and ATIP3 mRNA in normal and cancerous prostatic cell lines and provide further information on normal human prostate biopsy material using QPCR.

## 2. Results and Discussion

### 2.1. Results

#### 2.1.1. AT_2_-Receptor, ATIP1 and 3 mRNA Expression in Normal and Cancerous Human Prostatic Tissue and Cell Lines

The expression of ATIP1 and ATIP3 mRNA in RWPE1 normal and one androgen dependent (LNCaP) and two androgen independent (PC3 and DU145) prostate cancer cell lines was determined and compared (Table 1). In addition, we examined ATIP1 and ATIP3 expression in mRNA generated from normal human prostate tissue and prostate tissues containing moderately- and poorly-differentiated prostate cancer.

ATIP1 mRNA in the cell lines, was slightly higher (approximately 1.7-fold) in the LNCaP cells and significantly higher in DU145 cells (approximately 3.2-fold, P < 0.01), compared with RWPE1 normal prostatic cells whereas, its expression was approximately 2.3-fold lower in PC3 cells. In normal human tissues, ATIP1 mRNA was present and approximately 1.6-fold higher in moderately-differentiated prostate cancer biopsies and significantly higher in poorly-differentiated prostate cancer samples (approximately 3.7-fold, P < 0.05) compared with normal prostatic tissue (Table 1).

For ATIP3 mRNA, all three prostate cancer cell lines displayed significantly higher ATIP3 mRNA expression than RWPE1 cells (13.0 to 78.8-fold higher, P < 0.01). In moderately-differentiated cancer biopsies there was a trend for higher ATIP3 mRNA expression (2.0-fold increase) and significantly higher expression in poorly-differentiated prostate cancer (4.6-fold increase, P < 0.01) compared with normal prostatic tissues (Table 1).

Interestingly, the magnitude of the increase in mRNA expression in both cancer cell lines and human cancer compared with normal samples was greater for ATIP3 than for ATIP1. In the cell lines AT_2_-receptor mRNA was significantly higher in all three prostate cancer cell lines compared to RWPE1 cells. The fold-increases in expression compared to RWPE1 cells were 4.3, 4.6 and 12.1 for the LNCaP, PC3 and DU145 cells, respectively (Table 1). By contrast in human normal and prostate cancer biopsies, although there was a trend for higher AT_2_-receptor mRNA levels in cancer compared to normal tissues, there was no significant difference between the levels of mRNA present.

#### 2.1.2. Immunohistochemical Study of ATIP Expression in Prostate Cancer Cell Lines

**Figure 1.**
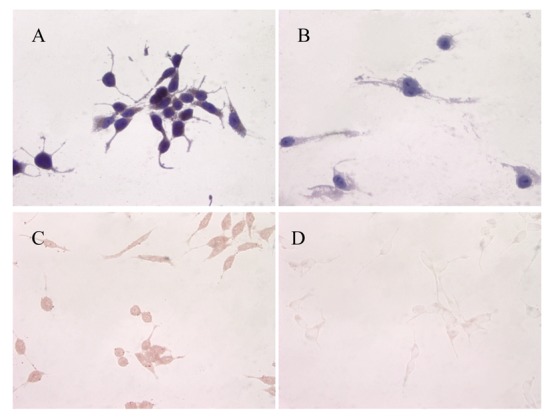
Immunolocalization of ATIP in LNCaP cells (**A** and **B**) with haematoxylin counter-stain (**A**) and without counter-stain (**C**); (**B** and **D**) serial cover slips pre-absorbed with ATIP antigen with (**B**) and without (**D**) haematoxylin counter-stain respectively. Images were taken at 400× magnification.

**Figure 2.**
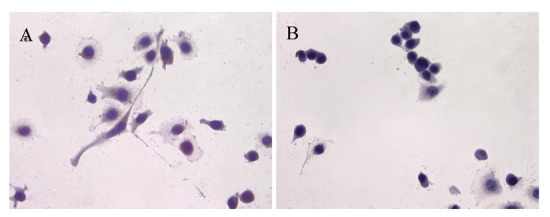

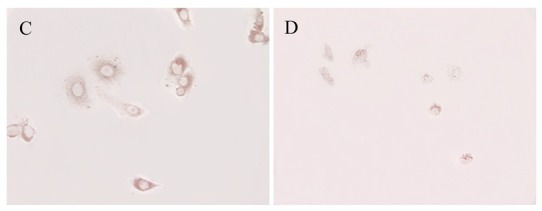
Immunolocalization of ATIP in PC3 cells (**A** and **C**) with haematoxylin counter-stain (**A**) and without counter-stain (**C**); (**B** and **D**) serial cover slips pre-absorbed with ATIP antigen with (**B**) and without (**D**) haematoxylin counter-stain respectively. Images were taken at 400× magnification.

ATIP protein expression was examined in fixed whole LNCaP and PC3 cells where it was localized to the cytoplasms of both cell lines (Figures 1 and 2). There was no staining (negative-control) in prostate cancer cells when the ATIP anti-body was pre-absorbed with ATIP antigen (Figures 1B,D and 2B,D).

#### 2.1.3. Immunohistochemical Studies in Paraffin Embedded Human Prostate Cancer Tissues

**Figure 3.**
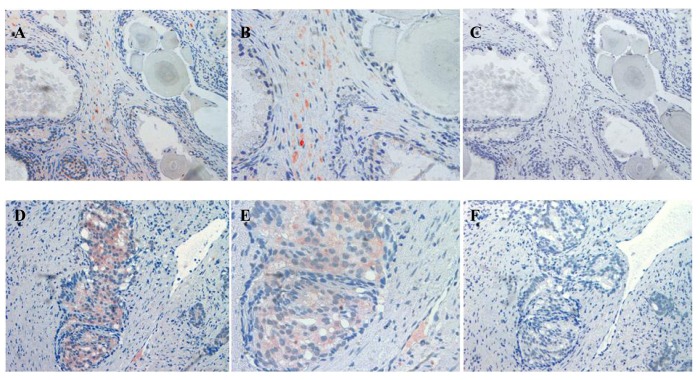
Immunolocalization of ATIP staining in stroma surrounding BPH (**A** and **B**) and HGPIN (**D** and **E**) gland in paraffin embedded tissue sections. Serial sections showing an absence of staining when the ATIP antibody was pre-absorbed (**C** and **F**) with ATIP antigen. Images were taken at (**A**, **C**, **D** and **F**) 200× and (**B** and **E**) 400× magnification.

**Figure 4.**
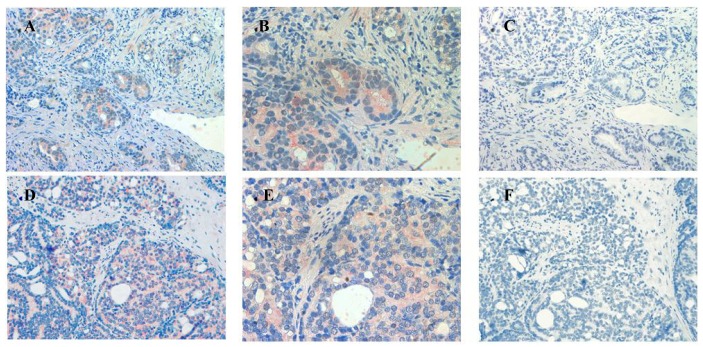

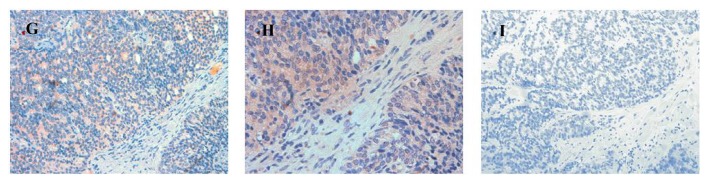
Immunolocalization of ATIP staining in well-differentiated (**A** and **B**), moderately-differentiated (**D** and **E**) and poorly-differentiated (**G** and **H**) prostate cancer in paraffin embedded tissue sections. Serial sections (**C**, **F** and **I**) showing little or no staining when the ATIP antibody was pre-absorbed with ATIP antigen. Images were taken at (**A**, **C**, **D**, **F**, **G** and **I**) 200× and (**B**, **E** and **H**) 400× magnification.

The localization of ATIP staining in the various developmental stages of human prostate cancer is shown in Figures 3 and 4. As only a small number of samples (n = 8–10) were examined for each group a qualitative analysis was performed to determine either the presence or absence of ATIP immunostaining in BPH, HGPIN, malignant tissues and the surrounding stroma (Table 2). ATIP immunoreactivity was readily identified in the stromal smooth muscle cells in BPH tissue, whereas low or undetectable levels were present in the basal or columnar epithelial cell layers that comprised the acini of BPH tissues (Figure 3B). In contrast in glands containing HGPIN, ATIP immunostaining was observed consistently in neoplastic epithelial cells and adjacent stroma, but not in the basal cell layer (Figure 3E).

In sections containing well-, moderately- and poorly-differentiated cancer, ATIP staining was almost always present in the malignant epithelial cells (Table 2, Figures 4B,E,H). By contrast, while immunostaining was present in stroma it was less visible than in HGPIN and tended to be less visible with progression of the malignancy. This may in part reflect diminishing amounts of stroma with loss of differentiation.

Little or no ATIP staining was detected in prostate tissues in negative control experiments, in which the primary antibody was pre-absorbed overnight with the ATIP antigen or when the primary antibody was replaced with PBS (Figures 4C,F,I**)**.

### 2.2. Discussion

This paper describes and provides some qualitative data on the expression and localization of ATIP protein in human prostate biopsies and *in vitro* models representing early (LNCaP cells) and late stage (PC3 cells) prostate cancer using an anti-ATIP antibody.

In addition, we have examined and compared ATIP1 and ATIP3 mRNA expression, the predominantly expressed ATIP isoforms in prostate [7], as well as AT_2_-receptor mRNA in RWPE1 normal prostate cells and three prostate cancer cell lines and also mRNA generated from normal human prostate tissue and biopsies containing moderate and high grade prostate cancer.

Previously, ATIP protein has been identified in the mitochondria of MIA PaCa-2 pancreatic carcinoma cells and HepG2 hepatocellular carcinoma cells [11,12], as well as in the Golgi matrix of COS-7 cells [6]. As a first step we investigated the cellular distribution of ATIP protein, using an anti-ATIP antibody that identifies all five ATIP isoforms [5], in cellular models of early (LNCaP) and late stage human prostate cancer (PC3). Like Ang II [13], ATIP staining was identified in the cytoplasms of both cell lines examined (LNCaP and PC3) using immunohistochemical techniques (Figures 1 and 2).

Our findings identifying the presence of ATIP in human biopsies taken from patients undergoing surgery for prostate cancer are quite novel. Although ATIP immunostaining was low or not detected in either the basal or columnar epithelial cell layers surrounding BPH glands, it was routinely observed in the neoplastic epithelial cells of HGPIN and in the cytoplasms of the malignant cells in all grades of prostate cancer (Figures 3 and 4). HGPIN is considered to be a precursor to prostate cancer [14,15] as it possesses most of the phenotypic, biochemical and genetic changes of cancer without invasion of the basement membrane of the acini [16]. The up-regulation of ATIP in neoplastic cells in HGPIN and its continued presence in cancer adds further support to the notion that malignant prostatic cells arise from the neoplastic epithelial cells of HGPIN [13].

In addition, the consistent appearance of ATIP staining in the precancerous epithelial cells and its relative absence in normal epithelial cells strongly suggest that re-expression or up-regulation of ATIP occurs at an early phase in the malignant process and that ATIP up-regulation may be an early indicator of malignancy. AT_2_-receptor re-expression has already been described in pathological conditions such as myocardial infarction [17,18] and wound healing [19] where it is thought to play an important role in differentiation and injury response [17,19]. The up-regulation of ATIP may also play an important role in prostate cancer and facilitate the anti-growth action of the AT_2_-receptor as an adaptive response to the excessive growth associated with early malignancy.

Previously, we have examined the correlation between ATIP1 mRNA expression and rate of proliferation in relatively slow growing androgen-dependent prostate cancer cells, LNCaP, and fast growing androgen-independent PC3 prostate cancer cells [10]. We hypothesized at the time that ATIP expression maybe inversely correlated with rate of proliferation in the two prostate cancer cell lines as similar relationships had been identified in both pancreatic and breast cancers [9,12].

On this occasion in addition to LNCaP and PC3 cells we examined the normal prostatic cell line, RWPE1, and another malignant cell line DU145. ATIP1 mRNA expression was higher in the LNCaP and DU145 cells compared to the normal cells, whereas PC3 expressed lower than normal ATIP1 mRNA levels. For ATIP3 mRNA, all three cancer cell lines expressed higher levels than the RWPE1 cells. These results suggest that neoplastic cells derived from prostate epithelial cells generally show an increased expression of both ATIP isomers and the results are consistent with our results in human malignancies (Table 1). The reason for low ATIP1 mRNA levels in PC3 cells is uncertain but this cell line also demonstrates low levels of functional AT_1_-receptors [2,4] and it is tempting to speculate that the two findings are related.

By contrast, ATIP3 levels were elevated in all three human prostate cancer cell lines and in human prostate cancer biopsies. Moreover, ATIP3 mRNA was re-expressed to higher levels than ATIP1. At this time the role of ATIP3 in prostate cancer is unknown; however, its role in breast cancer has been identified and it appears to slow mitosis and inhibit cell proliferation [9]; therefore, its function appears to be similar to that of ATIP1 [5,10,12]. This suggestion received support in an *in vitro* study where we used siRNA techniques to knock-down both ATIP1 and ATIP3 expression in LNCaP and PC3 cells using silencing probes that targeted the interacting domain that is present within all ATIP isoforms [10]. These studies indicated that the combined effect of reducing ATIP1 and ATIP3 mRNA expression in these cells resulted in an increase in the growth-promoting effects of EGF in LNCaP cells and the basal rate of growth in PC3 cells, further suggesting that ATIP3 may have a similar function in both prostate cancer and breast cancer.

Our studies have focused on normal and malignant epithelial cells; however, there is also evidence that Ang II via stimulation of AT_1_- and AT_2_-receptors may also have a role in mitogenesis within the stromal compartment of the prostate. AT_1_- and AT_2_-receptor mRNA and AT_1_- but not AT_2_-receptor protein have been identified in primary cultures of human prostatic stromal (hPS) cells [20] and in human prostatic stromal biopsies [21]. The absence of AT_2_-receptor protein in the later study is not surprising as it may result from the inherent difficulties in generating antibodies for seven transmemebrane receptors [22,23] and we are yet to find an AT_2_-receptor antibody that reliably identifies this protein in tissue lysates or biopsies (data not shown). Wennemuth *et al.* indicated that Ang II-induced activation of AT_1_-receptors in hPS cells resulted in an increase in intracellular calcium ion concentration and cell proliferation [21] possibly by transactivation of EGF-receptors, ErbB1 and Erb2 [20]. In the present immunohistochemical studies, ATIP protein was identified in the stroma surrounding the acini containing BPH, HGPIN and all grades of prostate cancer (Table 2), suggesting that ATIP may have a role in the Ang II mediated growth pathways previously identified in these stromal regions of the prostate. However, the function of ATIP in prostatic stromal cells remains to be elucidated.

The relevance of the ATIP/AT_2_-receptor interaction in malignant prostatic epithelial cells is more clearcut. Our earlier *in vitro* studies clearly indicate that in spite of the presence of relatively low levels of AT_2_-receptor mRNA, activation of the AT_2_-receptor significantly inhibited EGF-induced LNCaP and PC3 cell growth [2]; in the absence of AT_2_-receptor activation, ATIP mRNA expression decreased when the cells were stimulated with EGF [10]; and that AT_2_-receptor activation inhibited the EGF-induced down-regulation of ATIP mRNA. These studies, in combination with the work described previously in this section, indicate that EGF may stimulate prostatic cell growth, at least in part, by reducing the levels of ATIP present in the cells and that activation of the AT_2_-receptor may prevent this down-regulation from occurring, thus suggesting that an important interaction exists between ATIP and the AT_2_-receptor in mediating prostate cancer cell growth.

In addition, in the present studies the mRNA results in the cell lines indicate that, like ATIP mRNA expression, there is also an up-regulation of the AT_2_-receptor in prostate cancer and therefore a positive correlation between ATIP and AT_2_-receptor mRNA expression exists (Table 1**)**. However in the human tissues, although there was a trend for higher AT_2_-receptor mRNA expression in the cancer biopsies compared to the normal tissue, this relationship between the AT_2_-receptor and ATIP was not substantiated as there was no significant difference between the AT_2_-receptor levels in the human biopsies. This difference may in part be explained by the relative difficulty in detecting AT_2_-receptor mRNA compared to ATIP in both the cell lines and the tissues. In addition, it must be noted that the tissue mRNA is generated from a heterogeneous population of cells, unlike the cell line studies, which includes both malignant epithelial and normal stromal cells and this may significantly reduce the sensitivity of the assay system, thus making detection of changes in mRNA for the AT_2_-receptor in malignant cells more difficult. Unfortunately, in the absence of a reliable antibody for the AT_2_-receptor it is also difficult to determine whether there is any change in the localization of the AT_2_-receptor in malignant prostatic cells as it has been previously reported that ATIP levels significantly affect AT_2_-receptor expression at the plasma membrane in N1E-115 cells; with a decrease in ATIP expression resulting in a reduction of AT_2_-receptors at the cell surface [6]. If the converse is also true, and an up-regulation of ATIP results in increased AT_2_-receptor expression at the cell surface then this pathway may become more easily activated, even in the absence of changes in AT_2_-receptor mRNA levels. However, this exciting possibility remains to be investigated in prostate cancer cells.

## 3. Experimental Section

### 3.1. Cell Culture

Three human prostate cancer cell lines, DU145 [24], PC3 [24,25] and LNCaP [26] were grown in RPMI1640 media supplemented with 2 mM glutamine and 10% FCS. RWPE1, normal human prostate cells, were grown in keratinocyte serum free medium supplemented with 0.05 mg/mL bovine pituitary extract and 5 ng/mL EGF. All cell lines were maintained at 37 °C in a humidified atmosphere of 5% CO_2_/95% air.

### 3.2. AT_2_-Receptor, ATIP1 and ATIP3 mRNA Expression in Cell Lines

Primers and TaqMan probes for the AT_2_-receptor, ATIP1 and ATIP3 were designed by Ms Josefa Pete (Baker Institute, Melbourne, Australia) with the help of Primer Express v2.0 (Applied Biosystems, Carlsbad, CA, USA). Cells were seeded and cultured overnight in 12-well plates so that, on the following day the cells were approximately 70% confluent and were assumed to be growing at an exponential rate. Total RNA was then isolated from the cells as previously described [2]. Samples of cDNA (n = 4–6) were analyzed for ATIP expression using an Applied Biosystems 7500 Real-time PCR system according to our previously published method using VIC labeled 18sRNA (Applied Biosystems) as an internal control [2].

### 3.3. Anti-ATIPAntibody

ATIP expression was studied using a rabbit polyclonal anti-ATIP antibody (Institute Cochin, Paris, France) that identifies all five ATIP isoforms and which was made by sub-cloning the 354 bp ATIP-ID cDNA fragment into the pRESTA vector (Invitrogen, Carlsbad, CA, USA) [5].

### 3.4. Immunohistochemical Detection of ATIP in Human Prostate Cancer Cell Lines

Glass cover slips (2 cm × 2 cm) were sterilized and dried. PC3 and LNCaP cells were plated at 2 × 10^5^ cells/well in a 6 well plate, containing a sterilized cover slip in RPMI 1640 supplemented with 10% FCS and 2 mM L-glutamine and then incubated overnight at 37 °C/5% CO_2_.

The following day, the cover slips were removed and washed in PBS. Cells were then fixed in ice cold 100% methanol, prior to incubation in PBS containing 0.1% Triton ×100. Cover slips were washed with PBS, and then incubated with blocking solution (10% horse serum, 0.3% Triton ×100 and 3% BSA in PBS). Following further washing in PBS, cover slips were incubated overnight at 4 °C with anti-ATIP primary antibody (1:2,000 dilution for LNCaP and 1:3,000 dilution for PC3), in the presence or absence of a 1:10 dilution of ATIP antigen.

The next morning, cover slips were washed in PBS prior to a 60 minutes incubation with a biotin-free peroxidase labeled polymer conjugated to goat anti-rabbit secondary antibody (Envision^+^ system, DakoCytomation, Carpinteria, CA, USA). Finally, immunoreactivity was visualized by incubating with AEC (DakoCytomation) for 5 minutes at 37 °C, yielding a red end-product. Some of the sections were counter-stained with haematoxylin and Scott's tap water before being mounted in DAKO faramount aqueous mounting solution (DakoCytomation) for histological examination.

### 3.5. Human Prostate Cancer Tissues

The use of human prostate tissues in this study was approved by the Austin Health Human Research Ethics Committee (Heidelberg, Victoria, Australia, Approval No. H2009/03430). All tissues described were examined and graded histologically by an expect pathologist from the Department of Anatomical Pathology, A&RMC (Melbourne, Australia) to determine the disease state of the tissue according to the Gleason Grading system.

### 3.6. Fresh Frozen Normal Human Tissues

Normal prostate biopsies obtained post-mortem from eight male subjects aged 19 to 28 years were snap frozen with isopentane and dry ice. Prostate histology was confirmed as normal in each case.

### 3.7. QPCR for AT_2_-Receptor, ATIP1 and ATIP3 mRNA Expression in Fresh Frozen Prostate Cancer Biopsies

Fresh frozen mRNA was kindly provided by the Victorian Cancer Biobank. The biopsies included 11 containing moderately-differentiated prostate cancer (Gleason grades 5, 6 and 7) and 9 containing poorly-differentiated prostate cancer (Gleason grades 8, 9 and 10). AT_2_-receptor, ATIP1 and ATIP3 RNA were detected using the same methods and primers used to identify ATIP expression in the cell lines. Unfortunately at the time of writing, no samples corresponding to well-differentiated prostate cancer were available from the Biobank.

### 3.8. Immunohistochemical Detection of ATIP in Paraffin-Embedded Biopsies from Prostate Cancer Patients

Sections were taken from the biopsies of 10 subjects and all had regions of BPH, high grade prostatic intraepithelial neoplasia (HGPIN) and well-, moderately- and poorly-differentiated prostate cancer. Immunohistochemistry was performed according to the previously described methods [13]. Briefly, sections were de-paraffinized with xylene for 10 minutes and sequentially rehydrated in graded ethanol (100% and 95%) for 6 minutes. Antigen retrieval was performed using 10 mM citrate buffer (0.38 g citric acid and 0.24 g sodium citrate, pH 6.0) in dH_2_O for 2 minutes in microwave at high setting, and a further 7½ minutes set to medium-high. The solution was then allowed to cool for 20 minutes before the tissues were washed three times with dH_2_O and then treated with 3% H_2_O_2_-methanol for 10 minutes to remove endogenous peroxidase activity. Background binding was blocked using 5% skim milk in PBS for 10 minutes prior to incubation with 1:400 dilution of anti-ATIP antibody diluted in 1% BSA/PBS, for 25 minutes at room temperature. This was followed by 1 h incubation with a biotin-free peroxidase labeled polymer conjugated to goat anti-rabbit secondary antibody (Envision^+^ system, DakoCytomation). Finally, immunoreactivity was visualized with AEC chromogen for 7½ minutes at 37 °C yielding a red/brown end-product. The sections were counter-stained with haematoxylin and Scott's tap water before being mounted in DAKO faramount aqueous mounting solution for histological examination. Serial control sections were also examined where the primary ATIP antibody was replaced by PBS or the primary antibody had been pre-absorbed with a 1:10 dilution of ATIP antigen overnight at 4 °C.

### 3.9. Identification of Immunostaining

ATIP immunostaining in cell lines and paraffin-embedded tissue sections was examined using the Microcomputer Image Device (MCID)-M2 program (Image Research Incorporated, St. Catherine's, ON, Canada). Pictures were taken at 100×, 200× and 400× magnification of randomly chosen fields immuno-stained with the ATIP polyclonal antibody. For each prostate section, single fields containing BPH, HGPIN, well-differentiated, moderately-differentiated and poorly-differentiated cancer, were photographed. Qualitative analysis of the presence of ATIP was performed for the resulting 8–10 fields in each category (BPH, HGPIN, well-differentiated, moderately-differentiated and poorly-differentiated) in two key areas; the epithelial cells within the acini and the stroma. A score of “1” or “0”, respectively, was given if ATIP immunostaining was present or absent in the corresponding area.

### 3.10. Statistical Analysis

Real-time PCR results were analyzed for statistical significance by ANOVA or Student's *t*-test using Graphpad Instat ^®^ v3.06 (Graphpad Software Inc, La Jolla, CA, USA). Data are expressed as the mean ± SEM. The level of significance was set at P < 0.05, P < 0.01 and P < 0.001.

## 4. Conclusions

ATIP expression has been identified in human biopsies and *in vitro* models of early and late stage prostate cancer and, in general, prostate cancer is associated with increases in ATIP1 and ATIP3 mRNA expression, compared to normal tissues and cell lines.

The data further demonstrate that there is a re-expression of ATIP1 and ATIP3 in HGPIN and levels in neoplastic cells rise as the malignancy progresses to a less differentiated form. The progressive increase in the rate of growth of the poorly differentiated cancers in the presence of high levels of ATIP suggests that there may be dysfunction in the AT_2_-receptor/ATIP signaling pathway.

However, our earlier work [10] suggests that transfection and significant over-expression of ATIP1 has the ability to slow prostate cancer cell growth rate and the identification of ATIP/AT_2_-receptors in both normal and malignant prostatic biopsies, as shown in the present studies, indicate that this may be a potential therapeutic pathway for, not only, prostate cancer, but other prostatic diseases characterized by excessive cell growth.

## Figures and Tables

**Table 1.** Expression of ATIP1, ATIP3 and AT_2_-receptor mRNA in human prostate cell lines and tissues.

		**ATIP1** ΔCT	±SEM	Fold-Δ ATIP1 ^3^	**ATIP3** ΔCT	±SEM	Fold-Δ ATIP3 ^3^	**AT_2_-R** ΔCT	±SEM	Fold-Δ AT_2_-R ^3^
*Human cell lines*										
RWPE1	18.5		±0.3	1.0	20.6	±0.5	1.0	27.4	±0.2	1.0
LNCaP	17.7		±0.3	1.7	15.3	±0.3 **	39.4	25.3	±0.2 *	4.3
PC3	19.7		±0.2	0.4	16.9	±0.4 **	13.0	25.2	±0.3 **	4.6
DU145	16.8		±0.5 **	3.2	14.3	±0.4 **	78.8	23.8	±0.6 **	12.1
*Human Tissues*										
Normal	17.9		±0.5	1.0	17.8	±0.4	1.0	26.2	±0.4	1.0
Moderate ^1^	17.2		±0.4	1.6	16.8	±0.4	2.0	25.5	±0.3	1.6
Poor ^2^	16.0		±0.4 #	3.7	15.6	±0.2 ## *	4.6	25.6	±0.5	1.5

For ΔCT values the higher the number the lower the level of mRNA present. Each value represents the mean ± SEM of 4-11 independent mRNA samples.

1 Moderate-Gleason Grades 5–7;

2 Poor-Gleason Grades 8–10;

3 Fold-Δ—Fold-difference in mRNA expression to RWPE1 cells for the human cell lines and fold-difference to normal tissue for human biopsies. Fold-differences have been rounded to one decimal place. AT_2_-R—AT_2_-receptor.

** significantly higher expression than RWPE1 cells P < 0.01, ANOVA—Dunnett Multiple Comparison Test compared to normal cells (RWPE1);

# and ## significantly higher expression than normal prostate tissue P < 0.05 and P < 0.01, respectively, and

* significantly higher than moderately-differentiated, ANOVA—Bonferroni Multiple Comparisons Test—All pairs.

**Table 2.** Results of immunohistochemical studies for ATIP expression in paraffin-embedded human prostate sections expressed as percentage of tissues positive for ATIP staining.

**Area**	**BPH**	**HGPIN**	**Well %**	**Moderate**	**Poor**
Epithelial cells within gland	10 ± 0.0	100 ± 0.0	50 ± 0.1	100 ± 0.0	100 ± 0.0
Surrounding Stroma	100 ± 0.0	90 ± 0.1	50 ± 0.2	50 ± 0.2	50 ± 0.1

Values represent the % fields positive for ATIP immunostaining in the corresponding area of prostate stage as classified using the Gleason Grading System. 1 = ATIP present; 0 = ATIP not present. N = 8 independent samples for each classification, except for BPH where n = 10.
